# Systematics of *Parnassia* (Celastraceae) inferred from plastid and nuclear DNA sequence data

**DOI:** 10.3897/phytokeys.273.182792

**Published:** 2026-04-23

**Authors:** Wen-Xuan Zhang, Hong-Tao Li, Jin-Mei Lu, Ding Wu, De-Zhu Li

**Affiliations:** 1 Germplasm Bank of Wild Species & Yunnan Key Laboratory of Crop Wild Relatives Omics, Kunming Institute of Botany, Chinese Academy of Sciences, Kunming, Yunnan 650201, China Kunming Institute of Botany, Chinese Academy of Sciences Kunming China; 2 School of Tropical Agriculture and Forestry, Hainan University, Danzhou，Hainan 571737, China Shandong Agricultural University Tai’an China; 3 Center for Interdisciplinary Biodiversity Research & College of Forestry, Shandong Agricultural University, Tai’an, Shandong 271000, China Hainan University Danzhou China

**Keywords:** Character evolution, Chloroplast DNA, ITS, Molecular phylogeny, *
Parnassia
*

## Abstract

We presented a comprehensive phylogenetic analysis of *Parnassia* covering approximately 80% of recognized species. By integrating five plastid markers (*rbcL*, *matK*, *rpl32*-*trnL*, *trnT*-*trnL* and *trnL*-*trnF*) with nuclear marker ITS sequences and applying both maximum parsimony and Bayesian inference approaches, we reconstructed robust phylogenetic relationships within the genus. We presented notable differences between molecular phylogeny and the established sectional classification. While *Parnassia* sect. *Fimbripetalum* emerged as monophyletic with strong support, *P.* sect. *Saxifragastrum*, *P.* sect. *Cladoparnassis*, *P.* sect. *Nectaroquinquelobos*, and *P.* sect. *Allolobos* were resolved as paraphyletic. The monotypic *P.* sect. *Nectarobilobos* represented a distinct evolutionary lineage, whereas the species-rich *P.* sect. *Nectarotrilobos* was dispersed across multiple clades. Notably, three eastern Himalayan species (*P. faberi*, *P. esquirolii* and *P. labiata*) formed a well-defined clade with highly reduced staminodia. This study provided a critical molecular framework for reevaluating morphological evolution and revising the infrageneric classification of *Parnassia*.

## Introduction

The genus *Parnassia* L. (Celastraceae) comprises 50–70 species of perennial herbs predominantly distributed across arctic and temperate regions of the Northern Hemisphere (Phillips 1982; Hultgård 1987; Ku 1987; Wu et al. 2003; Simmons 2004; Wu 2005; Shu 2017). The genus is readily identified by its basal leaf rosettes, elongated peduncles bearing solitary flowers, pentamerous floral organization, and the distinctive presence of staminodia opposite each petal (Nekrassova 1917; Handel-Mazzetti 1941; Phillips 1982; Ku 1987; Gu and Hultgård 2001; Simmons 2004; Shu and Zhang 2017). Mountains of Southwest China and the Himalaya harbor centers of diversity, with more than 30 endemic species (Hultgård 1987; Wu et al. 2003; Shu et al. 2017; Dai et al. 2021, 2023). In contrast, North America possesses only approximately 10 species, including the widespread circumpolar *Parnassia palustris* and the Siberian-North American *Parnassia kotzebuei* (Hultgård 1987; Bonnin et al. 2002; Borgen and Hultgård 2003; Weber 2003).

Since its establishment by Linnaeus in 1753, the systematic position of *Parnassia* has been debated and historically placed in various families, notably Saxifragaceae*sensu lato* (Hooker and Thomson 1858; Dandy 1927; Engler 1930, 1964; Thorne 1976; Bohm et al. 1986; Ku 1995), with other proposed affinities. Contemporary molecular systematics, however, firmly supports its inclusion within Celastraceae (APG IV 2016).

Within this established familial framework, molecular phylogenetic studies consistently identify the monotypic genus *Lepuropetalon* Elliott as sister to *Parnassia* (Simmons et al. 2001; Simmons 2004; Zhang and Simmons 2006; Yang et al. 2012), a relationship corroborated by both vegetative and floral morphology (e.g., Murbeck 1918; Spongberg 1972; Gornall and Al-Shammary 1998; Simmons 2004). Broad-scale molecular analyses consistently resolve the [*Lepuropetalon* + *Parnassia*] clade as sister to Celastraceae (e.g., Soltis and Soltis 1997, 2000; Soltis et al. 2011), with more focused family-level studies positioning this clade within an early-diverging Celastraceae lineage with weak (Simmons et al. 2001; Bacon et al. 2016) to high statistical support (Zhang and Simmons 2006; Xia et al. 2022).

**Figure 1.**
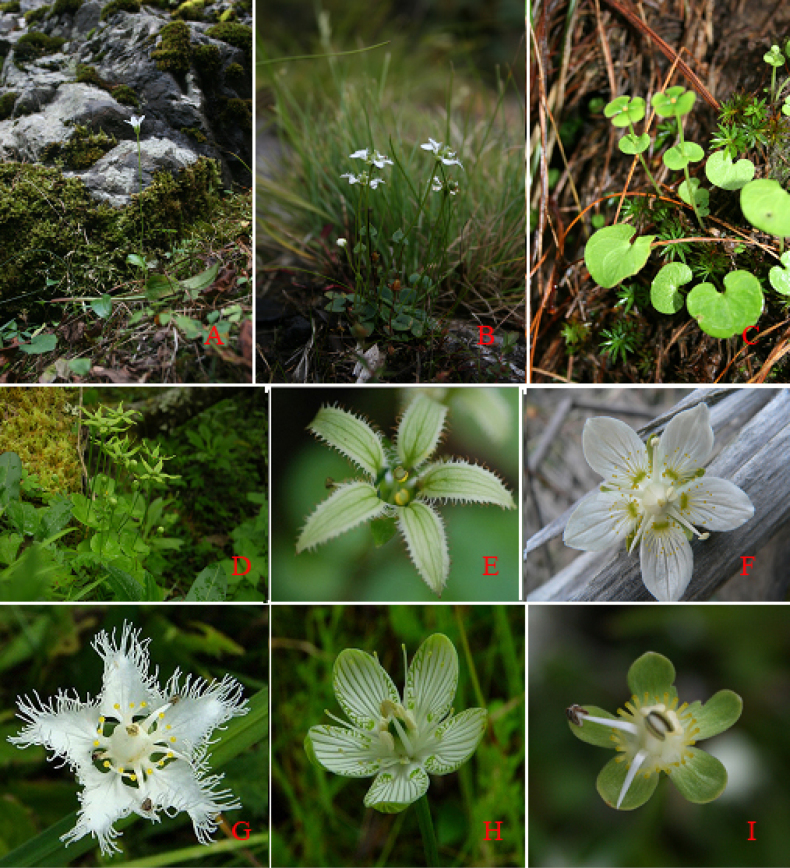
Habitat and petal and staminodial characteristics of *Parnassia*. **A–D**. Natural habitats: **A**. *P. laxmanni* (*P.* sect. *Nectarotrilobos*); **B**. *P. farreri* (*P.* sect. *Odontohymen*); **C**. *P. tenella* (*P.* sect. *Saxifragastrum*); **D**. *P. longipetala* (*P.* sect. *Saxifragastrum*); **E–I**, Petal margins and staminodial morphology: **E**. *P. cooperi* (*P.* sect. *Nectarotrilobos*); **F**. *P. palustris* (*P.* sect. *Parnassia*); **G**. *P. foliosa* (*P.* sect. *Fimbripetalum*); **H**. *P. grandifolia* (*P.* sect. *Parnassia*); **I**. *P. wightiana* (*P.* sect. *Allolobos*). Photos by Ding Wu and Shu- Dong Zhang.

Infrageneric classification presents equally complex challenges. Drude (1875) established an infrageneric taxonomic system of *Parnassia* and recognized four sections: *P.* sect. *Nectarotrilobos*, *P.* sect. *Fimbripetalum*, *P.* sect. *Saxifragastrum*, and *P.* sect. *Nectarodroson*, which was later expanded by Engler (1930) to include *P.* sect. *Cladoparnassia*. While most species of *P.* sect. *Nectarodroson* are from North America with the exception of *P. palustris* and *P. kotzebuei*, species of other sections in the genus are concentrated in the Altai and Himalayan regions of Asia. Asian taxa exhibit significant morphological diversity (Handel-Mazzetti 1941; Gu and Hultgård 2001), with species identification relying heavily on staminodial characteristics, petal margins, and cauline leaf number (Fig. 1). Phillips (1982) subsequently divided Drude’s *P.* sect. *Nectarodroson* (≡ *P.* sect. *Parnassia*) into two smaller sections (*P.* sect. *Parnassia* and *P.* sect. *Longiloba*). Built on the taxonomic framework of Drude (1875), Ku’s (1987) treatment of Chinese species added four additional sections, viz. *P.* sect. *Odontohymen*, *P.* sect. *Nectarobilobos*, *P.* sect. *Allolobos*, and *P.* sect. *Nectaroquinquelobos*, based primarily on the staminodium traits, petal entire or fimbriate, and number of cauline leaf. Wu et al. (2003) proposed a modified classification system based on Ku’s (1987) framework. Thus, current infrageneric taxonomy primarily reflects the systems of Drude and Engler’s (1930) in Table 1.

**Table 1.** Comparative overview major infrageneric classifications in *Parnassia* (number of species in parentheses).

Drude (1875) & Engler (1930)	Ku (1987)	Wu et al. (2003)
Sect. *Parnassia* (13)	Sect. *Parnassia* (1)	Sect. *Parnassia* (1)
Sect. *Fimbripetalum* (6)	Sect. *Cladoparnassia* (2)	Sect. *Allolobos* (10)
Sect. *Nectarotrilobos* (23)	Sect. *Fimbripetalum* (4)	Sect. *Fimbripetalum* (4)
Sect. *Saxifragastrum* (9)	Sect. *Nectarobilobos* (1)	Sect. *Nectarobilobos* (1)
Sect. *Cladoparnassia* (1)	Sect. *Nectaroquinquelobos* (4)	Sect. *Xiphosandra* (3)
	Sect. *Nectarotrilobos* (30)	Sect. *Odontohymen* (2)
	Subsect. *Amblysandra* (27)	Sect. *Nectarotrilobos* (28)
	Subsect. *Xiphosandra* (3)	Sect. *Cladoparnassia* (3)
	Sect. *Odontohymen* (2)	Sect. *Saxifragastrum* (8)
	Sect. *Saxifragastrum* (10)	
	Sect. *Allolobos* (5)	

Molecular systematics has rapidly advanced our understanding of *Parnassia* phylogeny. Zhang and Simmons (2006) confirmed the monophyly of the genus but with limited intrageneric resolution due to sparse taxon sampling. Our previous work (Yang et al. 2012) enhanced geographical representation and explored DNA barcoding applications. Xia et al. (2022) provided insights into chloroplast genomics and codon usage analysis in *Parnassia*. Nevertheless, a comprehensive phylogenetic framework incorporating North American representatives remains lacking. Here, we employ an expanded dataset comprising five cpDNA regions (*rbcL*, *matK*, *rpl32*-*trnL*, *trnT*-*trnL*, *trnL*-*trnF*) and nrDNA ITS. Increased taxon and character sampling for *Parnassia* is expected to (1) reconstruct a robust molecular phylogeny of *Parnassia*; (2) critically evaluate existing infrageneric classifications; (3) identify morphological synapomorphies for major clades; and (4) establish a phylogenetically-informed taxonomic framework.

## Materials and methods

### Taxon sampling

Our sampling strategy encompassed 45 *Parnassia* species, representing approximately 80% of the genus and all sections recognized by Ku (1987). Most material was collected from wild populations in China, supplemented by herbarium specimens (K, E, US, BH). Based on strong evidence supporting *Lepuropetalon* as sister to *Parnassia* (e.g., Simmons et al. 2001; Zhang and Simmons 2006; Soltis et al. 2011) and indications of relationship to *Mortonia*, *Microtropis*, *Pottingeria*, *Quetzalia*, and *Zinowiewia* (Zhang and Simmons 2006; Simmons et al. 2012), we selected five species from these genera as outgroup taxa. Voucher details and GenBank accessions are presented in Suppl. material 1.

### Molecular marker amplifications

We extracted total DNA from silica gel-dried leaves and herbarium specimens using a modified CTAB protocol (Doyle and Doyle 1987). DNA quality was verified by 1.5% agarose gel electrophoresis. We amplified and sequenced the ITS region with primers ITS5/ITS4 (White et al. 1990), the *trnL*-*trnF* region (including the *trnL* intron) with primers c/f (Taberlet et al. 1991), the *trnT*-*trnL* intergenic spacer with primers a/b (Taberlet et al. 1991), and the *rbcL* gene with primers *rbcL* a-f (Kress and Erickson 2007)/724R (Fay et al. 1997). The *matK* gene was amplified using Xf/5r primers (Ford et al. 2009).

Polymerase chain reaction (PCR) amplifications were conducted in a 20 μL reaction mixture containing 1 × *Taq* buffer [50 mM (NH_4_)_2_SO_4_; 75 mM Tris-HCl (pH 8.3); 50 mM KCl; 0.001% gelatin]; 2.5 mM MgCl_2_, 0.4 mM of dNTPs, 0.5 μM of each primer, 1.0 U of *Taq* DNA Polymerase (TaKaRa Biotechnology Co. Ltd., Dalian, China), and 1 μL of genomic DNA (25–30 ng). Thermal cycling conditions comprised: an initial template denaturation at 94 °C for 5 min, 35 cycles of 30 seconds denaturation at 94 °C, 1 minute primer annealing at 52 °C, 1.5 min extension at 72 °C, with a final extension of 8 min at 72 °C. Ampliﬁcation products were puriﬁed using the QIAquick Gel Extraction Kit. Purified PCR products were sequenced bidirectionally using the PCR primers on an ABI 3730 DNA Sequencer (Applied Biosystems, Inc.).

### Phylogenetic analyses

Sequence alignments were performed using MAFFT v7.409 (Katoh and Standley 2013) and manually refined in BioEdit Sequence Alignment Editor v.7.0.0. We assessed dataset congruence using the incongruence length difference (ILD) test in PAUP*4.0b10 (Swofford 2002) following Wang et al. (2004). Based on the ILD test results, we analyzed three datasets separately: nrDNA ITS, cpDNA regions (*rbcL*, *matK*, *rpl32*-*trnL*, *trnT*-*trnL*, *trnL*-*trnF*), and a combined 5-gene dataset. Phylogenetic reconstruction employed maximum parsimony (MP), maximum likelihood (ML) and Bayesian inference (BI) approaches.

We determined the optimal substitution model (GTR+G+Γ) using ModelFinder v2.1.6 (Kalyaanamoorthy 2017) under the Akaike information criterion (AIC). ML analyses were conducted with RAxML 8.2.X (Stamatakis 2014) using 1,000 rapid bootstrap replicates. BI analyses were performed with MrBayes v 3.2.7a (Ronquist et al. 2012) via the CIPRES Science Gateway V 3.3, nodes with posterior probabilities (PP) > 0.95 were considered significant.

## Results

### Sequence characteristics

The ILD test detected no significant incongruence among the five cpDNA regions (P > 0.01). The aligned ITS region spanned 738 bp (including adjacent regions of the 18S and 26S genes sequences), with the 5.8S subunit exhibiting high conservation (165 bp). The combined chloroplast dataset (4049 bp) contained 787 variable sites (19.4%), including 568 parsimony-informative characters (14.0%). The *trnT*-*trnL* (605 bp) and *matK* (861 bp) markers showed the highest variability (227 and 254 variable sites, and 190 and 170 parsimony-informative sites, respectively), while *rbcL* (711 bp) and *rpl32*-*trnL* (836 bp) were more conserved (75 and 126 variable sites, 50 and 78 parsimony-informative characters, respectively). The *trnL*-*trnF* intergenic spacer exhibited complete congruence with *trnT*-*trnL*, with all 105 variable sites being parsimony-informative characters.

### Plastid phylogeny

**Figure 2.**
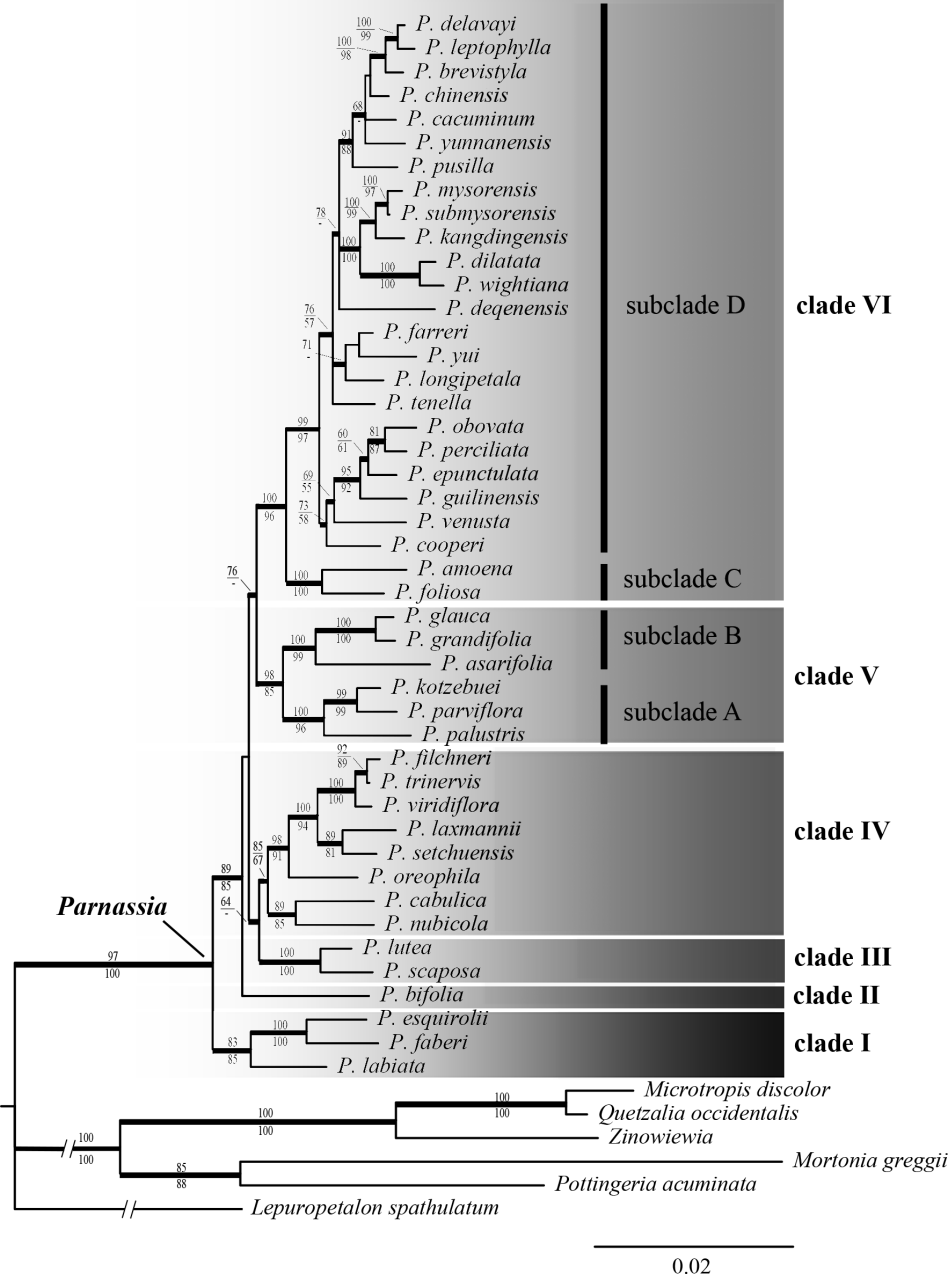
Fifty percent majority-rule consensus tree from the BI analysis of combined cpDNA data. Topologies from ML and MP analyses were congruent with the BI tree. Bayesian posterior probability values ≥ 0.90 are indicated by bold branches. MP/ML bootstrap values ≥ 50% are shown above/below branches, respectively, otherwise, a hyphen (-) is used. For *Zinowiewia*, combined sequences from multiple species are represented at the generic level.

Combined cpDNA analyses strongly supported the monophyly of *Parnassia* (BS=100, PP=1.0), resolving six major clades (Fig. 2). Clade I (BS=83, PP=1.0) comprised three Chinese species (*P. faberi*, *P. esquirolii*, *P. labiata*). Clade II contained only *P. bifolia*, positioned basal to a large clade encompassing clades III-VI. Clade III (BS=100, PP=1.0) included *P. lutea* and *P. scaposa*. Clade IV (BS=85, PP=0.99) united part of *P.* sect. *Nectarotrilobos* characterized by ovate-oblong basal leaves and three- lobed staminodia. Clade V (BS=98, PP=1.0) contained primarily North American species divided into two subclades (A: BS=100, PP=1.0; B: BS=100, PP=1.0), though *P. kotzebuei* extends into Siberia and *P. palustris* exhibits circumboreal distribution.

Clade VI (BS=100, PP=1.0), the largest and probably the most recently diversified, included nearly half of all sampled species, predominantly from the Himalayan- Hengduan Mountains (except *P. procul* in North Sumatra). Within this clade, *P. foliosa* and *P. amoena* formed a subclade sister to the remaining taxa. Despite limited resolution within clade VI, five of the six subclades were corroborated by unambiguous morphological synapomorphies.

### Nuclear ribosomal ITS phylogeny

**Figure 3.**
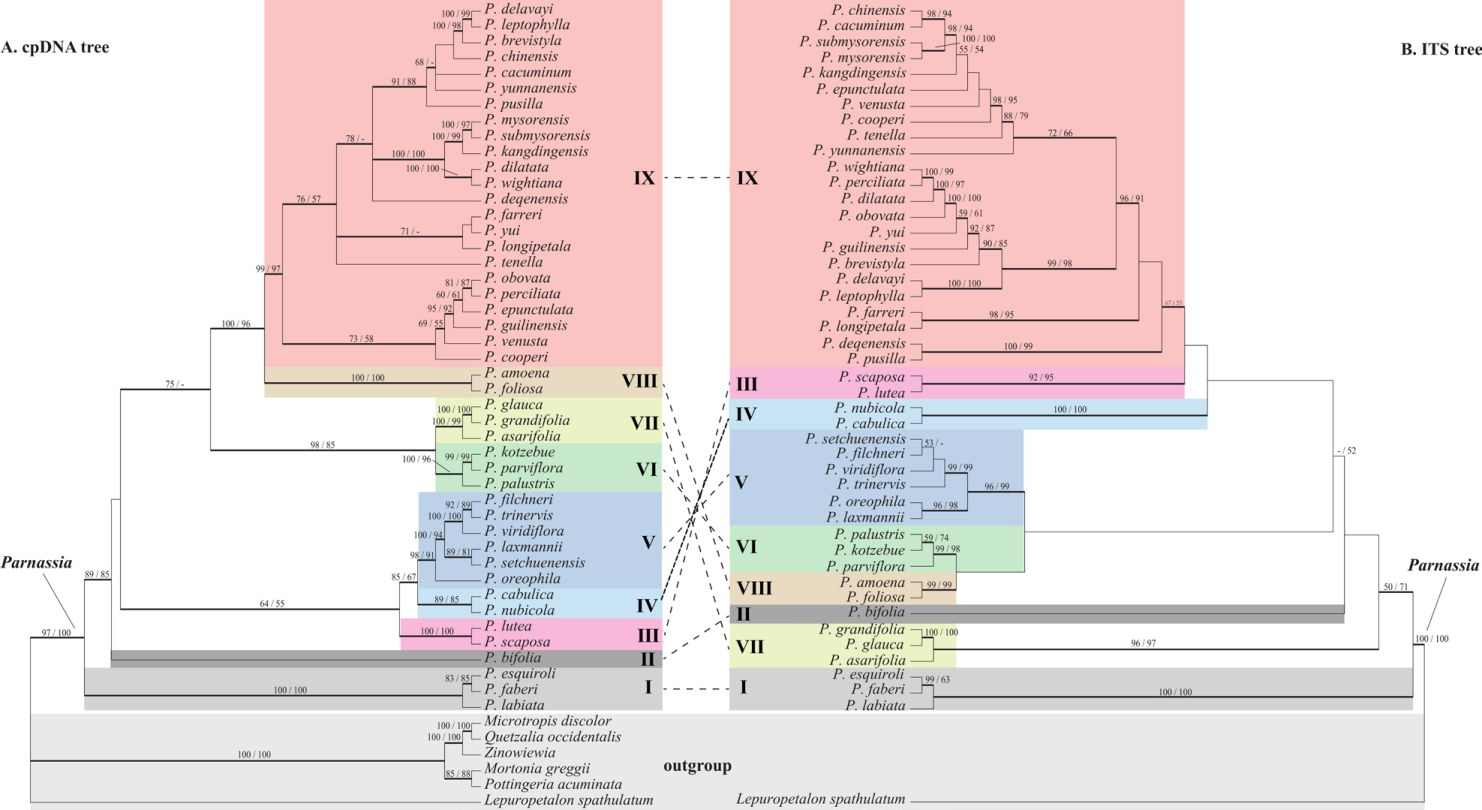
Phylogenetic relationships in *Parnassia* based on cpDNA (**A**) and nrDNA (**B**) data. Branch numbers indicate ML/MP bootstrap values. Dashed boxed highlight topological conflicts between ML trees. All branch lengths are significant (*P*=0.01).

The nrDNA phylogeny employed *Lepuropetalon spathulatum* as the sole outgroup, accommodating high substitution rates in Celastraceae (Bai et al. 2015). MP analysis yielded multiple equally parsimonious trees differing mainly in clade placement (Fig. 3). Three species of *P.* sect. *Longiloba* Phillips (*P. asarifolia*, *P. grandifolia*, *P. glauca*) formed a highly supported clade (Clade VII, Fig. 3B), as did *P. palustris*, *P. kotzebuei*, and *P. parviflora* (Clade VI, Fig. 3B). Several clades showed complete concordance between plastid and nuclear topologies (e.g., Clade IX). Notably, *P.* sect. *Fimbripetalum* demonstrated strong monophyly (Clade VIII, Fig. 3B), while North American species formed a distinct clade, sister to *P. bifolia* (Clade II, Fig. 3B). The largest clade was well resolved but inconsistent support at internal nodes. *P. farreri* showed affinity to members of *P.* sect. *Saxifragastrum* (*P. longipetala*, *P. tenella*, *P. yunnanensis*), with which it shares staminodial morphology.

Six species from Drude’s *P.* sect. *Nectarotrilobos* (equivalent to Ku’s *P.* sect. *Nectarotrilobos* ser. *Nectarotrilobos*) formed a well-supported clade (BS=99, PP=1.0), sharing ovate-oblong basal leaves with truncate or cuneate bases. In particular, those species with simple three lobed staminodia, this similarity to Asian species may be the result of homoplastic evolution. The low sequence divergence within the Hengduan Mountains clade suggests either recent rapid ecological radiation or conservation of plesiomorphic floral traits, especially staminodial and petal morphology.

## Discussion

### Reconciliation of molecular phylogeny with classification systems

The intricate taxonomic history of *Parnassia* largely stems from extreme morphological plasticity in staminodia, petal margins and floral pigmentation (e.g., Phillips 1982; Ku 1987; Wu et al. 2003; Wu 2005). Our cpDNA phylogeny identifies six well-supported clades, each definable by morphological or biogeographical features. Comparison with Ku’s (1987) system reveals both concordance and conflict signals: (1) Engler’s (1930) *P.* sect. *Cladoparnassia* and Ku’s (1987) *P.* sect. *Saxifragastrum* ser. *Rosulatae* form monophyletic clade I; (2) Ku’s (1987) monotypic *P.* sect. *Nectarobilobos* corresponds to clade II; (3) *P. lutea* and *P. scaposa* (Ku’s *P.* sect. *Nectarotrilobos* subsect. *Amblysandra* ser. *Luteae*) comprise clade III; (4) a Drude’s (1875) *P.* sect. *Nectarotrilobos* subgroup with subtle morphological variation (Handel-Mazzetti; Phillips 1982; Ku 1987) constitutes clade IV; (5) Phillips’s (1982) sections, *P.* sect. *Parnassia* and *P.* sect. *Longiloba* form monophyletic clade V; and (6) the diverse clade VI amalgamates elements from Drude’s (1875) sections *P.* sect *Saxifragastrum*, *P.* sect. *Fimbripetalum* and *P.* sect *Nectarotrilobos*, along with Ku’s (1987) sections *P.* sect. *Odontohymen*, *P.* sect. *Allolobos*, and *P.* sect. *Nectaroquinquelobos*. These data illustrate the value of chloroplast gene sequence data in resolving genetic, and hence phylogenetic, relationships among members of the most taxonomically complex groups.

Members of each respective section share similar morphological traits or geographical distributions. Ku (1987) correctly recognized the affinity between bractless species *P. lutea* and *P. scaposa* (*P.* subsect. *Amblysandra* ser. *Luteae*). However, the limited molecular divergence within *P.* sect *Nectarotrilobos* – coupled with its remarkable morphological variation—indicates rapid radiation with extensive ecological diversification and speciation. The morphological diversity encompassed by this group is so great that subsequent workers have provided substantially modified concepts of relationships (Wu et al. 2003; Wu 2005). Because of the number of species representing many of the intermediates between extremes, it is not possible to adequately subdivide the section into smaller groups in a formal classification.

### Evolutionary implications of morphological characters

Current *Parnassia* classification reflects traditional interpretation of evolutionary trends in the floral morphology, emphasizing staminodial elaboration and biogeography (Ku 1987). Our results, consistent with previous studies (Phillips 1982; Zhang and Simmons 2006), demonstrate that staminodial morphology—the primary character in traditional classifications—exhibits considerable homoplasy. The two groups in North America are shown here to be highly distinct. *Parnassia* sect. *Nectarotrilobos* was the most species rich section and represented the most diversified clade in the genus. *Parnassia* species in the Sino-Himalayan region, particularly the Hengduan Mountains (Ku 1987; Wu et al. 2003; Wu 2005; Wu et al. 2009), exhibit greater variability. Eastern Asia and North America represent two centers of diversity for this genus. These findings advance the taxonomy of *Parnassia* and provide a foundation for studying its evolutionary diversity.

Traditional infrageneric classifications by Drude (1875), based mainly on staminodium shape and petal margin, were not supported by molecular data. Sections characterized by long-fimbriate petals (*P.* sect. *Fimbripetalum*), mallet-shaped staminodes (*P.* sect. *Saxifragastrum*), or prominently 3–7 lobed staminodes (*P.* sect. *Nectarotrilobos*) were not monophyletic. The close relationship between *P. faberi*, *P. esquirolii* and *P. labiata* is not reflected in their classification. The classification of the Asian species presents rather more serious difficulties. Several groups are relatively distinct. *P. foliosa* and *P. amoena*, readily recognized by the presence of 3 to 9 cauline leaves on the peduncle and elaborate fimbriate petals, are members of Drude’s (1875) section *Fimbripetalum*. Although *P. noemiae* was not included in this analysis, it is apparent from the description that it also belongs in section *Fimbripetalum*, having character states very similar to *P. foliosa* and *P. amoena*. Phylogenetic results suggest that mallet-shaped staminodes present the ancestral condition, while lobed or globose- tipped staminodes forms evolved independently. Without substantial evidence regarding relative ancestral or derived character states, it is difficult to evaluate which is the most primitive section.

Several nodes—particularly within *P.* sect. *Nectarotrilobos*— exhibited low support (BS < 80%, PP < 0.95), likely reflecting recent rapid radiation in the Himalaya – Hengduan region, incomplete lineage sorting, and insufficient phylogenetic signal. Limited marker variation and ongoing gene flow or hybridization may further blur genetic resolution. We observed topological conflicts between plastid and nuclear datasets. For infrageneric revision, we prioritized the cpDNA phylogeny due to its higher nodal support and topological stability. Plastid genomes offer advantages for resolving deep relationships given their uniparental inheritance and low recombination rates. ITS conflicts likely reflect faster evolution, incomplete lineage sorting, or historical hybridization— exemplified by putative hybrid *P. yui*, where cpDNA indicates *P. farreri* and *P. longipetala* as maternal donors. While cpDNA provides a robust classification framework, we acknowledge these conflicts and emphasize the need for future phylogenomic studies.

Although molecular data do not support staminode-based classifications, these results suggest that incomplete lineage sorting, hybridization, or gene introgression events may have frequently occurred among sympatric species. For instance, fringed petal margins evolved independently in *P. foliosa* and *P. farreri*, reflecting similar pollinator-mediated selection. Taxonomic difficulties in *Parnassia* also stem from two distinct morphological issues. First, characters once considered diagnostic have proven unreliable upon broader sampling. The prolongation of the anther connective, for example, was traditionally used to distinguish *P. delavayi*, *P. brevistyla*, and *P. leptophylla* (Franchet 1897). However, Yu et al. (2018) demonstrated that this trait varies continuously among populations and formally treated the latter two names as synonyms of *P. delavayi*. Second, continuously varying quantitative traits have frequently been partitioned into multiple segregate species, as seen in the P. wittiana complex (including *P. gansuensis*, *P. yiliangensis*, and *P. monochorifolia*; Wu et al. 2004; Wu 2005; Wang et al. 2018).

For the putative hybrid P. yui, chloroplast data indicate *P. farreri* and *P. longipetala* as maternal donors, though paternal ancestry remains unresolved.

The most important contribution of this study is not the proposal of a modified classification, but rather the evidence for interspecific relationships that can guide further investigations within the genus. Also distinct is the group composed of *P. esquirolii*, *P. faberi* and *P. labiata*. This section, *P.* sect. *Cladoparnassia*, is distinguished by its small habit and flowers, and simple undivided or slightly transversely notched, club-shaped staminodia. *Lepuropetalon spathulatum*, the sister genus to *Parnassia*, was used as the outgroup in the parsimony analysis. Morphological comparisons between *Lepuropetalon* and the *P. faberi* group reveal shared traits such as reduced stature and simplified floral architecture, yet their phylogenetic distance precludes direct inference of ancestral character states without formal ancestral state reconstruction (Simmons et al. 2001; Zhang and Simmons 2006). Future studies employing explicit character mapping and broader outgroup sampling are needed to rigorously assess whether rosette leaves represent a plesiomorphic condition in *Parnassia*. Furthermore, topological conflicts between the plastid and nuclear ITS phylogenies were observed in the relationships of certain taxa within Clades V and VI (e.g., the *P. palustris* complex and *P. farreri*), suggesting that these lineages may have experienced hybridization, introgression, or incomplete lineage sorting. It should be noted that the Sanger sequencing markers employed in this study provide insufficient informative sites to resolve recent rapid radiations in the Hengduan Mountains region, contributing to low nodal support within Clade VI. Future studies integrating phylogenomic data are needed to further clarify these relationships.

## Revised infrageneric classification

The center of diversity for *Parnassia* is in central Asia, especially the Himalayan axis where the majority of the species occur. Our molecular results largely contradict Ku’s (1987) sectional boundaries but support the foundational framework of Drude and Engler. We propose the following revised infrageneric classification of *Parnassia* reflecting phylogenetic relationships; this revised classification is primarily based on the phylogenetic relationships revealed by the cpDNA tree (Fig. 2):

### 1. [clade I] Parnassia sect. Cladoparnassia

Taxon classificationPlantaeCelastralesCelastraceae

Engl. in Engl. u Prantl, Nat. Pflanzenfam. Aufl. 2, 18a: 182. 1930

78907068-DD82-5A0D-B311-4DF2112AA07E

= Parnassia sect. Saxifragastrum Drude ser. Rosulatae T.C.Ku in Bull. Bot. Res. North- East. For. Univ. 7 (1): 28. 1987. – Type: P. esquirolii Lévl.

#### Type.

*P. faberi* Oliv.

#### Diagnosis.

The typical characteristic of this clade is that the basal leaves are numerous, densely clustered in a rosette. The staminodes are structurally simple, usually unlobed at the apex or only bilobed in a lip-like form.

### 2. [clade II] Parnassia sect. Nectarobilobos

Taxon classificationPlantaeCelastralesCelastraceae

T.C.Ku in Bull. Bot. Res., Harbin. 7 (1): 46. 1987

84A3D6FB-68F5-5414-821F-D37953ADBAA4

#### Type.

*P. bifolia* Nekr.

#### Diagnosis.

This clade is monospecific, containing only *P. bifolia*, which is distinguished by having two cauline leaves and staminodes that are flat and bilobed at the apex.

### 3. [clade III] Parnassia sect. Luteae

Taxon classificationPlantaeCelastralesCelastraceae

(T.C.Ku) D.Wu & D.Z.Li stat. nov.

293CBF51-C537-5E3B-BCC8-D339A2486E51

urn:lsid:ipni.org:names:77378964-1

≡ Parnassia sect. Nectarotrilobos Drude subsect. Amblysandra (Franch.) T.C.Ku ser. Luteae T.C.Ku in Bull. Bot. Res., Harbin. 7 (1): 31. 1987.

#### Type.

*P. lutea* Batal.

#### Diagnosis.

A prominent feature of this clade is the absence of cauline leaves. The basal leaves are ovate to oblong, with a truncate or cuneate-decurrent base. The staminodes are flat and trilobed.

### 4. [clade IV] Parnassia sect. Longifolia

Taxon classificationPlantaeCelastralesCelastraceae

D.Wu & D.Z.Li sect. nov.

22D992EB-C71E-5865-8859-0D4E1BC0C04D

urn:lsid:ipni.org:names:77378965-1

#### Type.

*P. oreophila* Hance.

#### Diagnosis.

The morphological characteristics of this new section include the presence of one cauline leaf. The basal leaves are similar in morphology to those of Clade III, being ovate to oblong with a truncate or decurrent base. The staminodes are also flat and trilobed.

### 5. [clade V] Parnassia sect. Parnassia

Taxon classificationPlantaeCelastralesCelastraceae

1588B851-A3E7-5667-B150-A52625C6ECE5

≡ Parnassia sect. Nectarodroson Drude in Linnaea 39: 301. 1875 = Parnassia sect. Amblysandra Franch. in Bull. Soc. Bot. France 44: 251. 1897.

#### Type.

*P. palustris* L.

#### Diagnosis.

The basal leaves are usually cordate or reniform, with a cordate base. The staminodes are deeply lobed, reaching half or more of their length.

### 5a. [subclade A] Parnassia sect. Parnassia subsect. Parnassia

Taxon classificationPlantaeCelastralesCelastraceae

F3D47126-E915-5678-BCA3-7106E052F31C

#### Type.

*P. palustris* L.

#### Diagnosis.

The typical features include staminodes that are flat with as many as 5–30 lobes. The seed coat is often sac-like.

### 5b. [subclade B] Parnassia sect. Parnassia subsect. Longiloba

Taxon classificationPlantaeCelastralesCelastraceae

D.Wu & D. Z.Li subsect. nov.

AA980301-BF70-5908-AC3A-8AB2F83AB28B

urn:lsid:ipni.org:names:77378966-1

#### Type.

*P. grandifolia* DC.

#### Diagnosis.

The staminodes are branched (not flat), usually with three lobes (rarely 4–5). The petals are entire. There is typically one cauline leaf. *P.* sect. *Longiloba* was first recognized by Phillips (1982) but neither effectively nor validly published; it is validated here as a new subsection.

### 6. [clade VI] Parnassia sect. Nectarotrilobos

Taxon classificationPlantaeCelastralesCelastraceae

Drude in Linnaea 39: 302. 1875

B410EE1E-15A4-552B-B457-A050454DF976

≡ Parnassia sect. Nectarotrilobos Drude subsect. Amblysandra (Franch.) T.C.Ku in Bull. Bot. Res., Harbin. 7 (1): 31. 1987 ≡ Parnassia sect. Nectarotrilobos Drude subsect. Amblysandra (Franch.) T.C.Ku ser. Nectarotrilobos T.C.Ku in Bull. Bot. Res., Harbin. 7 (1): 32. 1987 = Parnassia sect. Nectarotrilobos Drude subsect. Amblysandra (Franch.) T.C.Ku ser. Lijiangenses T.C.Ku in Bull. Bot. Res., Harbin. 7 (1): 32. 1987.

#### Type.

*P. mysorensis* Heyne ex Wight & Arn.

#### Diagnosis.

The basal leaves are mainly cordate or reniform, with a cordate base. The staminodes are either unlobed or lobed to a depth not exceeding half their length. The seed coat is compact or slightly sac-like.

### 6a. [subclade C] Parnassia sect. Nectarotrilobos Drude subsect. Fimbripetalum

Taxon classificationPlantaeCelastralesCelastraceae

(Drude) D.Wu & D.Z.Li stat. nov.

658AC774-9E33-568B-982B-0142B0F7A478

urn:lsid:ipni.org:names:77378968-1

≡ Parnassia sect. Fimbripetalum Drude in Linnaea 39: 302. 1875 ≡ Parnassia sect. Fimbripetalum Drude ser. Fimbripetalum T.C.Ku in Bull. Bot. Res., Harbin. 7 (1): 51. 1987. **–** Type: P. foliosa Hook. f. et Thomson.
= Parnassia sect. Fimbripetalum Drude ser. Amoenae T.C.Ku in Bull. Bot. Res., Harbin. 7 (1): 51. 1987.

#### Type.

*P. amoena* Diels.

#### Diagnosis.

The petal margins bear fringed hairs. The plants often have three or more cauline leaves. The staminodes are branched.

### 6b. [subclade D] Parnassia sect. Nectarotrilobos Drude subsect. Nectarotrilobos

Taxon classificationPlantaeCelastralesCelastraceae

71DFA8E5-A21A-52B2-B434-FDFAE3B5224B

= Parnassia sect. Saxifragastrum Drude in Linnaea 39: 301. 1875 ≡ Parnassia sect. Saxifragastrum Drude ser. Saxifragastrum T.C.Ku in Bull. Bot. Res., Harbin. 7 (1): 22. 1987. – Type: P. tenella Hook. f. et Thomson.
= Parnassia sect. Odontohymen T.C.Ku in Bull. Bot. Res., Harbin. 7 (1): 29. 1987. – Type: P. farreri W.E.Evans
= Parnassia sect. Allolobos T.C.Ku in Bull. Bot. Res., Harbin. 7 (1): 46. 1987. – Type: P. wightiana Wall. ex Wight & Arn.
= Parnassia sect. Nectaroquinquelobos T.C.Ku in Bull. Bot. Res., Harbin. 7 (1): 52. 1987. – Type: P. perciliata Diels
= Parnassia sect. Xiphosandra Franch. in Bull. Soc. Bot. France 44: 262. 1897 ≡ Parnassia sect. Nectarotrilobos Drude subsect. Xiphosandra (Franch.) T.C.Ku in Bull. Bot. Res., Harbin. 7 (1): 44. 1987. – Type: P. delavayi Franch.

#### Type.

*P. mysorensis* Heyne ex Wight & Arn.

#### Diagnosis.

This subclade exhibits high morphological diversity and encompasses the principal lineages of Clade VI, excluding Subclade C (characterized by fringed petals and branched staminodes). Its core morphological variations are manifested in: staminodes that are club-shaped to columnar (corresponding to the former sect. *Saxifragastrum*), flat and trilobed (corresponding to the former sect. *Nectarotrilobos* s.str.), flat and pentalobed (corresponding to the former sect. *Allolobos*), or short-fan- shaped with apical teeth (corresponding to the former sect. *Odontohymen*). Additionally, lineages with significantly elongated stamen connectives (corresponding to the former sect. *Xiphosandra*) are also nested within it. Despite these pronounced morphological differences, particularly in staminode structure, phylogenetic analyses consistently recover these groups as a single, well-supported monophyletic clade.

## Supplementary Material

XML Treatment for Parnassia sect. Cladoparnassia

XML Treatment for Parnassia sect. Nectarobilobos

XML Treatment for Parnassia sect. Luteae

XML Treatment for Parnassia sect. Longifolia

XML Treatment for Parnassia sect. Parnassia

XML Treatment for Parnassia sect. Parnassia subsect. Parnassia

XML Treatment for Parnassia sect. Parnassia subsect. Longiloba

XML Treatment for Parnassia sect. Nectarotrilobos

XML Treatment for Parnassia sect. Nectarotrilobos Drude subsect. Fimbripetalum

XML Treatment for Parnassia sect. Nectarotrilobos Drude subsect. Nectarotrilobos

## Additional information

### Artificial Intelligence (AI) use

The authors accept full responsibility for the content of the manuscript, including the disclosure of any use of AI.

No AI tools were used in the preparation of this manuscript.

### Author ORCIDs

W.-X. Zhang https://orcid.org/0009-0005-8340-4311

H.-T. Li https://orcid.org/0000-0002-1290-0917

J.-M. Lu https://orcid.org/0000-0002-8728-8750

D. Wu https://orcid.org/0009-0000-5127-742X

D.-Z. Li https://orcid.org/0000-0002-4990-724X

### Data availability

The newly generated nucleotide sequences (plastid and ITS) have been deposited in the GenBank database. All data supporting the findings of this study are available within the manuscript and its supplementary material.

## Supplementary materials

Supplementary material 1 Voucher information and GenBank accession numbers for *Parnassia* and outgroup taxa sampled in this study https://doi.org/10.3897/phytokeys.273.182792.suppl1 Data type xlsx https://binary.pensoft.net/file/1605530This dataset is made available under the Open Database License (http://opendatacommons.org/licenses/odbl/1.0/). The Open Database License (ODbL) is a license agreement intended to allow users to freely share, modify, and use this Dataset while maintaining this same freedom for others, provided that the original source and author(s) are credited. Wen-Xuan Zhang, Hong-Tao Li, Jin-Mei Lu, Ding Wu, De-Zhu Li
